# Sexual orientation and gender identity and expression conversion exposure and their correlates among LGBTQI2+ persons in Québec, Canada

**DOI:** 10.1371/journal.pone.0265580

**Published:** 2022-04-06

**Authors:** Martin Blais, Fabio Cannas Aghedu, Florence Ashley, Mariia Samoilenko, Line Chamberland, Isabel Côté

**Affiliations:** 1 Research Chair in Sexual Diversity and Gender Plurality, Université du Québec à Montréal, Montréal, Québec, Canada; 2 Département de sexologie, Université du Québec à Montréal, Montréal, Québec, Canada; 3 Faculty of Law and Joint Centre for Bioethics, University of Toronto, Toronto, Ontario, Canada; 4 Département de travail social, Université du Québec en Outaouais, Gatineau, Québec, Canada; University of Perugia: Universita degli Studi di Perugia, ITALY

## Abstract

**Background:**

Despite greater acceptance of sexual and gender diversity and the scientific consensus that same-gender attraction, creative gender expression, and transness are not mental illnesses, LGBTQI2+ persons are still commonly told that they can or should change their sexual orientation, gender identity, or gender expression (SOGIE). The aim of this study was to describe the prevalence of SOGIE conversion efforts, including their sociodemographic correlates, among LGBTQI2+ persons.

**Methods:**

Using community-based sampling, we assessed SOGIE conversion attempts and involvement in conversion services of 3,261 LGBTQI2+ persons aged 18 years and older in Quebec, Canada.

**Results:**

A quarter of respondents experienced SOGIE conversion attempts, and fewer than 5% were involved in conversion services. Over half of those who were involved in SOGIE conversion services consented to them, but the services’ goals were made clear and explicit to only 55% and 30% of those who engaged in SO and GIE conversion, respectively. The results also suggest that family plays a key role in SOGIE conversion attempts and services utilization, and that indigenous, intersex, transgender, non-binary, and asexual persons, people of colour, as well as individuals whose sexual orientation is not monosexual (i.e., bisexual, pansexual) were more likely to have been exposed to conversion attempts and involved in conversion services.

**Conclusions:**

This study found that the prevalence of conversion efforts is substantial. Interventions to protect LGBTQI2+ people from such attempts should focus not only on legal bans, but also on supporting families who need to be counseled in accepting sexual and gender diversity. Health professionals need to be adequately trained in LGBTQI2+ affirmative approaches. Religious therapists should consult with colleagues and undergo supervision to ensure that their religious beliefs do not interfere with their practice.

## Introduction

Despite greater acceptance of sexual and gender diversity and the scientific consensus that same-gender attraction, creative gender expression, and transness are not mental illnesses, lesbian, gay, bisexual, trans, queer, intersex, two-spirit (LGBTQI2+) persons are still commonly told that they can or should change their sexual orientation (SO), gender identity (GI), or gender expression (GE). A recent study revealed that two-thirds of youths aged 13–24 years in the US reported that someone had tried to convince them to change their SOGIE [1]. Such pressures stem from the belief that it is best to be heterosexual and cisgender (i.e., not transgender) and that people should be changed when they do not conform to cisheterosexist norms. The concept of SOGIE change efforts has been coined to describe any direction or advice that intentionally delays or impedes self-acceptance of one’s sexual orientation, gender identity, or gender expression [2–4]. The 2020 edition of the Canadian *Sex Now Survey* revealed that about 20% of sexual minority men (gay, bisexual, transgender, Two-Spirit and queer) have been exposed to such efforts [5]. The US Transgender Survey found that 14% of respondents reported lifetime exposure to gender identity conversion efforts [6].

Conversion therapy refers to more sustained, structured, specific interventions aiming at changing, discouraging, or repressing SOGIE [2–4]. It relies on various techniques, inspired by psychotherapeutic, medical, or faith-based principles (e.g., talk therapy, aversion therapy, hormonotherapy, spiritual guidance) and takes place in various contexts ranging from private or public settings to “gay conversion camps” or religious institutions. Not only are they inefficient, scientifically unsound, and unethical, but they are also known to have adverse effects on survivors [6–8]. It is thus unsurprising that most psychological, medical, and sexual health professional associations have opposed SO conversion efforts for decades, and most of them are now adopting similar responses to GIE conversion efforts [9]. In December 2020, a ban on SOGIE conversion therapies has been adopted in Québec. This new law targets practices intended to “induce persons to change their sexual orientation, gender identity or gender expression or to repress non-heterosexual sexual behaviour” and applies regardless of age [10]. Violations are subject to heavy fines, professional discipline, and/or victim compensation. Federally, a criminal ban has been adopted by the Senate [11]. According to this new law, anyone who advertises, materially benefits from, or causes a person to undergo SOGIE conversion therapy would be liable to imprisonment for up to five years depending on the offence.

Professional associations’ statements are insufficient, as conversion experiences continue to be reported. In the US, about 4% of youths aged 13–24 years have undergone SOGI conversion therapy [7]. In Canada, three large, non-probabilistic studies have documented SOGI conversion experiences: the *Sex Now* survey 2011–2012 (N = 8,388) revealed that about 3.5% of Canadian sexual minority men (i.e., gay, bisexual, transgender, Two-Spirit and queer) experienced SO conversion therapy (4.2% in Quebec; [4]), the *Sex Now* survey 2019–2020 (N = 9,214) found that 9.9% of participants were exposed to SOGI conversion therapy [3]; and the *Trans Pulse Canada* survey (N = 2033) found that 11% of transgender and non-binary people had experienced conversion therapy [12].

### Survivor characteristics

In Canada, men who have sex with men (MSM) who have been exposed to SO or GI conversion efforts or practices were more likely to be gay (compared to bisexual), transgender and non-binary (compared to cisgender), “out” about their sexual orientation (relative to those who were not), younger, immigrants, and to earn an annual personal income of less than $30,000 (compared to those who earn $60,000 or more [3, 4]. The *TransPULSE* study found increasing rates of conversion therapy experiences with age [12]. In a US cohort of middle-aged and older MSM, Meanley et al. [13] found lower exposure to conversion therapy among MSM with any college education, but higher exposure among participants who enrolled in the studies post-2001 (compared to those enrolled pre-1987). Among US youths, Green et al. [7] found a higher prevalence of conversion experiences among gay and lesbian youths (relative to youths identifying as bisexual or as “something else”) and those from low-income families.

Conversion effort exposure appears to be evenly distributed before and after the age of 18 years. Societal, legal, and cultural homo- or trans-negativity is often endorsed by parents of LGBTQI2+ youths [14, 15], leading them to seek conversion efforts for their children. Furthermore, these beliefs may lead LGBTQI2+ child(ren) to believe that they would be more accepted if they were heterosexual and cisgender. Growing up with cultural and parental cisheteronormative values is thus likely to influence LGBTQI2+ persons to initiate, be exposed to, or participate in conversion efforts, whether voluntarily or not.

To explore such societal, legal, or cultural contexts, previous studies have relied on variables such as race/ethnicity, age cohort, religious affiliation, or family’s support of SOGIE. Salway et al. [4] found greater SO conversion therapy prevalence among Canadian Indigenous individuals and other racial minorities (relative to White men), but no differences were found between age groups. The study found that conversion efforts were far more common among trans respondents (12.1% versus 3.5%). Salway et al. [3] found that the prevalence of SOGI conversion therapy practices was higher among younger generations, immigrants, and racial/ethnic minorities. Meanley et al. [13] found a greater prevalence of these practices among US middle-aged and older Black MSM (both non-Hispanic and Hispanic) and those of other racial minority groups (compared to non-Hispanic White men), while Green et al. [7] found a higher prevalence among Hispanic/Latinx youths. In Ryan et al.’s [8] sample, respondents who were not born in the US were more likely than those born in the US to report having been taken to a therapist or religious leader by their parents to change their SO. Hypothesizing the role of more conservative values, Flores et al. [16] found lower support for banning conversion therapy among US racial and cultural minority groups. This conclusion supports the finding that ethnic minority parents report greater levels of homonegativity than ethnic majority parents [17]. Given the between-country variations in attitudes toward SOGIE, we can expect variations in exposure to conversion efforts depending on the country of birth.

Youth who underwent SO or SOGI conversion therapy are also more likely to come from religious families [8] or to have heard their parents (or caregivers) use religion to justify saying negative things about LGBTQ individuals [7]. Adamson et al. [18] found that, in their worldwide sample, about one-fourth of respondents who have been exposed to conversion efforts indicated that they had sought conversion therapy on their own, while the rest of the sample reported that this decision was beyond their control or made on their behalf by their family, religious leaders or community, school, or employer. They also found that most practitioners who led conversion therapy were mental health providers, followed by religious authorities or their associates.

While LGBTQI2+ people in Canada have been subjected to SOGIE conversion efforts, data are still scarce as most studies are limited to sexual minority men and specifically to SO conversion efforts. In Quebec, the only prevalence estimates available come from the *Sex Now* survey data, which found conversion therapy rates of 4.2% for SO [4] and 6.8% for SOGI [3] among Canadian sexual minority men. While little is known about conversion efforts among other sexual orientation groups (e.g., bisexual and pansexual individuals) and across genders and gender modalities (i.e., cisgender or transgender), rates of conversion efforts appear higher among transgender people [3, 4, 12, 19]. Moreover, as asexuality has only been recently recognised as different from sexual desire disorders [20], it is likely that persons describing their sexual orientation as asexual are more likely to have experienced sexual orientation conversion efforts or to have sought services to help them change. Also, to our knowledge, data on intersex persons’ experiences with gender identity assignment or modification are also scarce, though such experiences are likely as intersex variations are treated as a medical condition falling under sex/gender (re)assignment. Relying on a large, province-wide community-based survey, the current study describes the prevalence of SOGIE conversion attempts and involvement in conversion services, as well as their sociodemographic correlates, among LGBTQI2+ persons in Quebec.

## Method

### Participant recruitment

Data on SOGIE conversion experiences were collected as part of the Understanding the Inclusion and Exclusion of LGBTQ People (UNIE-LGBTQ) research project, which aimed to document events during which LGBTQI2+ people (aged 18 years and older) were demeaned, rejected and belittled, or deprived of the full extent of their rights in important life domains. Participants were recruited from September 2019 to August 2020 (before any legal ban on conversion therapy in Quebec or Canada) through the project’ and community partners’ communication channels (emails, listservs, the project website, Facebook pages and groups, Twitter, and LinkedIn), web and printed media, and word of mouth. The survey was administered online and was available in both French and English. Inclusion criteria were understanding French or English, being at least 18 years old, self-identify as LGBTQI2+, and live in the province of Quebec.

Over 6,000 persons accessed the online questionnaire, of which we retained only those who provided a valid Quebec postal code or whose IP address was located in the province (n = 6,095). Participants who did not provide sufficient data to confirm their eligibility or who did not meet the inclusion criteria were excluded (n = 1,115, of which 11 did not consent, 71 were younger than 18, and 85 were not LGBTQI2+). The final sample was composed of 4,980 participants. The present paper is based on the data of the 3,261 respondents who provided information on their exposure to SOGIE conversion attempts or their involvement in SOGIE conversion services. This study was approved by the Institutional Research Ethics Board of the Université du Québec à Montréal (Québec, Canada) (Protocol #2775).

### Measures

We assessed lifetime involvement in conversion therapy services and lifetime exposure to conversion attempts. Both were measured separately for SO and GIE, as social attitudes and professional guidelines toward sexual diversity and gender diversity are different. The four items used to assess these constructs, and their response options, are presented in Table 1. The research team created the survey questions based on the scientific literature and by consulting experts on conversion therapy and key informants from community-based organizations.

**Table 1:** Items measuring SOGIE conversion attempts and involvement in conversion services.

Variables	Questions		Response options
Involvement in Conversion Services	In your lifetime, have you been involved, voluntarily or otherwise, in services…	to change your sexual orientation or to avoid being or becoming gay, lesbian or bisexual?	Yes
			No
			Not applicable (coded as *No*)
		to avoid becoming or being trans, to change your gender identity or gender expression, or to help you conform to the sex or gender assigned to you at birth?	
Conversion Attempts	Among the following people, have specific people at any time tried to…	change your sexual orientation or prevent you from becoming gay, lesbian or bisexual?	No one
			Parent or family member (or their representatives)
			Conjugal partner or ex-partner
		prevent you from being or becoming trans, tried to change your gender identity or gender expression, or tried to make you conform to the sex or gender assigned to you at birth?	Friend
			Health professional (doctor, psychiatrist, psychologist, sexologist, etc.)
			Member of the clergy or of a religious group
			Other

Questions on lifetime involvement in conversion services were introduced by explicitly stating the aim of such services. Participants were provided with the following instructions: “The next questions ask about the contacts you may have had with services for which your aim was, for instance, *[see* Table 1 *for specific wording of the aim for SO and GIE]*. These services may have been provided by health professionals, spiritual or religious guides, or other types of people. These services may go by various names, including therapy or treatment (conversion, reparative, or corrective), special consultations, spiritual processes, healing or deliverance sessions, or other names you may be familiar with. Answer the following questions in relation to all the services you have been in contact with taken together, whether this was on your own initiative or upon someone else’s request (parent, partner, spiritual advisor, etc.)”. Contrary to these organized efforts, conversion attempts refer to any direction or advice to change someone’s SOGIE, make them conform to cisheterosexist norms, or to prevent them from becoming gay, lesbian, bisexual, or transgender.

We asked those who were involved in conversion services to provide information on their most recent experience: their age at the time, and the type of service provider (response options were: a doctor or psychiatrist; a psychologist; a sexologist; another type of therapist or psychotherapist; a member of the clergy, of a religious group, or of a church; no recollection of the person; other). We also inquired about the goals of the services. For SO conversion, the goals were: “to prevent you from being or becoming gay, lesbian, or bisexual”; “to change your sexual orientation (e.g., toward heterosexuality)”; and “to change how you express yourself in your body (your mannerisms, your ways of speaking, moving, walking, dressing, doing your hair, etc.)”. For GIE conversion, the possible goals were: “to prevent you from being or becoming trans”; “to change your gender identity (e.g., to become cisgender)”; and “to change how you express your gender identity with your body (your mannerisms, your ways of speaking, moving, walking, dressing, doing your hair, etc.)”. The four response anchors were dichotomized: not at all (coded 0); a bit (coded 0); somewhat (coded as 1); and a lot (coded as 1). Respondents also indicated whether they themselves, their parents (or their representatives), or someone else consented to these services and whether their conversion purposes were explicit from the beginning (response options: yes; no).

We also explored six potential motivations to seeking conversion services: 1) “I thought it would be easier for me and for my future if I tried”, 2) “I wanted to become [heterosexual, or cisgender] or to avoid becoming [gay, lesbian, or bisexual, or trans]”, 3) I was afraid of negative consequences if I refused to try (e.g., family rejection, refusal of care, termination of treatment), 4) “I felt that my loved ones would be happy if I did it”, 5) “I could not say no to the person or people who suggested it to me”, and 6) “They convinced me it was a good idea to try”. The response options ranged from 0 (Totally false) to 3 (Totally true).

Additional data on year of birth, intersex variation, sexual orientation, gender modality and identity, race/ethnicity, education, place of birth, household income, parents’ religious attendance, and perceived parents’ attributed importance to religious upbringing were also collected.

### Data analysis

Descriptive statistics were computed to summarize the sample’s characteristics. Continuous variables were presented as means and standard deviations, or as medians and intervals defined by the first and third quartiles. For dichotomous variables, their prevalence and 95% exact confidence intervals (CI) were calculated [21]. For categorical variables, we presented the proportion of each category and the corresponding 95% CI were calculated simultaneously for multinomial proportions [22]. Chi-square or Ficher exact tests were used to compare the distributions of the lifetime exposure to SOGIE conversion attempts and involvement in conversion services between cisgender LGBQ+ and trans participants. Crude Poisson regression with a robust error variance [23] was applied to assess the associations between lifetime exposure to SOGIE conversion attempts or service involvement and participant’s characteristics on the prevalence ratio (PR) scale. Analyses were performed using STATA 16.1 and SAS 9.4.

Missing data on the outcome variables followed a monotone pattern reflecting sections order in the online questionnaire, ranging from 34.5% (for conversion services involvement) to 39.7% (for conversion attempts). For both outcomes, weak associations [24, 25] were found between the presence of missing data and education (Cramer’s V between 0.12 and 0.13), and between the missingness in SOGIE conversion services involvement and birth cohort (Cramer’s V = 0.12), suggesting that missing data on the outcome variables were not completely random. Participants with a university degree and older participants were more likely to have completed the questionnaire. The percentage of missing values in the analytic sample was below 9% for most variables included in the present paper but exceeded 10% for two variables (parental religious attendance and attitudes). Statistical guidelines suggest that bias is negligeable with less than 10% missingness [26]. Missing values were not replaced. The significance level was set at *p* < 0.05.

## Results

### Participants

Table 2 presents the sample’s characteristics. While most participants were born after 1980, multiple birth cohorts were represented. Most participants described their sexual orientation as gay or lesbian (59%), bisexual (17%), or pansexual (10%). Over 80% of the sample was cisgender (43% women, 40% men), and 17% were transgender or non-binary (12% trans men and non-binary assigned female at birth, 5% trans women and non-binary persons assigned male at birth). Fourteen persons reported intersex variations. Most of the sample was white (89%) but included indigenous people (3%) and people of color (8%). Over half of participants reported a college or university degree (56%), most were born in Canada (87%), and were equally distributed across the four assessed household income brackets. About one-third of respondents reported that their parents never attended religious services and did not attribute any importance to religious upbringing.

**Table 2:** Sample characteristics.

Variable	N	%
**Birth Cohort**		
< 1955	180	5.52
1956–1970	425	13.03
1971–1980	387	11.87
1981–1990	829	25.42
> 1990	1440	44.16
**Sexual Orientation**		
Gay or Lesbian	1,932	59.25
Bisexual	537	16.47
Queer	243	7.45
Pansexual	340	10.43
Asexual	104	3.19
Other	105	3.22
**Gender Modality & Identity**		
Cisgender	2,705	82.95
Cisgender Women	1,400	42.93
Cisgender Men	1,305	40.02
Trans and non-binary	556	17.05
Trans men and non-binary AFAB	379	11.62
Trans women and non-binary AMAB	177	5.43
**Intersex Variation**		
No	3,237	99.26
Yes	14	0.43
Missing	10	0.31
**Race/Ethnicity**		
White	2,899	88.90
Indigenous	108	3.31
Racialized	254	7.79
**Education**		
< College Degree	1,392	42.69
College/University Degree	1,851	56.76
Missing	18	0.55
**Place of Birth**		
Abroad	434	13.31
Canada	2,826	86.66
Missing	1	0.03
**Household income**		
< 30,000	744	22.82
30,000–59,999	768	23.55
60,000–99,999	721	22.11
> 99,999	741	22.72
Missing	287	8.80
**Parents’ Religious Attendance**		
Never	1,117	34.25
1–2 times/year	731	22.42
> 3 times/year	205	6.29
> 1 /month	223	6.84
> 1/week	592	18.15
Missing	393	12.05
**Parents’ Attributed Importance to Religious Upbringing**		
Not at all	1,156	35.45
Not very	587	18.00
Somewhat	586	17.97
Very	409	12.54
Extremely	197	6.04
Missing	326	10.00

### Lifetime prevalence of SOGIE conversion attempt exposure and involvement in conversion services

Overall, 26.4% (95% CI, 24.8% to 28.0%) of respondents have experienced lifetime SOGIE conversion attempts or have been involved in conversion services (see Table 3). Cisgender sexual minority participants were more likely to have experienced conversion efforts targeting their SO (20.0%, 95% CI, 18.4 to 21.5) than their GIE (6.2%, 95% CI, 5.3 to 7.2), while trans participants were more likely to have been targeted for their GIE (41.9, 37.5 to 46.4) rather than their SO (25.6%, 95% CI, 21.8 to 29.7). Overall, trans participants were more likely to have been exposed to SOGIE conversion attempts and involved in SOGIE conversion services.

**Table 3:** Lifetime exposure to SOGIE conversion attempts and involvement in conversion services.

	Total	Cisgender LGBQ+ Participants	Trans Participants	*p*-value^1^
	n/N (%, 95% CI)			
Any Conversion Attempts or Services Involvement				
SO	630/3,013 (20.9, 19.5 to 22.4)	502/2,513 (20.0, 18.4 to 21.5)	128/500 (25.6, 21.8 to 29.7)	0.0047
GIE	363/2,999 (12.1, 11.0 to 13.3)	154/2,500 (6.2, 5.3 to 7.2)	209/499 (41.9, 37.5 to 46.4)	<0.0001
Any SO or GIE	799/3,031 (26.4, 24.8 to 28.0)	560/2,524 (22.19, 20.6 to 23.9)	239/507 (47.1, 42.3 to 51.6)	<0.0001
Conversion Attempts				
SO	598/3,008 (19.9, 18.5 to 21.4)	474/2,507 (18.9, 17.4 to 20.5)	124/501 (24.8, 21.0 to 28.8)	0.0028
GIE	345/2,995 (11.5, 10.4 to 12.7)	144/2,499 (5.8, 4.9 to 6.8)	201/496 (40.5, 36.2 to 45.0)	<0.0001
Any SO or GIE	757/3,018 (25.1, 23.5 to 26.7)	529/2,514 (21.0, 19.5 to 22.7)	228/504 (45.2, 40.8 to 49.7)	<0.0001
Involvement in Conversion Services				
SO	115/3,258 (3.5, 2.9 to 4.2)	102/2,703 (3.8, 3.1 to 4.6)	13/555 (2.3, 1.3 to 3.4)	0.0960
GIE	41/3,255 (1.3, 0.9 to 1.7)	13/2,699 (0.5, 0.3 to 0.8)	28/556 (5.0, 3.4 to 7.2)	<0.0001
SO or GIE	145/3,261 (4.4, 3.8 to 5.2)	110/2,705 (4.1, 3.4 to 4.9)	35/556 (6.3, 4.4 to 8.7)	0.0202

^1^ From Chi-square or Ficher exact tests to compare cisgender LGBQ+ and trans participants.

### SOGIE conversion attempts

Over two-thirds of respondents identified family members as responsible for the SOGIE conversion attempts (see Table 4), followed by friends and acquaintances, members of the clergy, and relationship (ex-)partners. Ten percent or less of participants identified healthcare professionals as responsible for such efforts. Trans participants were more likely than their cisgender LGBQ+ counterparts to have experienced GIE conversion attempts by friends or acquaintances (28.4%, 95% CI: 22.2% to 35.1% vs 15.3%, 95% CI: 9.8% to 22.2%), relationship (ex-)partner(s) (22.9%, 95% CI: 17.3% to 29.3% vs 11.1%, 95% CI: 6.5% to 17.4%), and healthcare professionals (12.9%, 95% CI: 8.6% to 18.4% vs 2.1%, 95% CI: 0.4% to 6.0%).

**Table 4:** Types of persons who have tried at any time to change participants’ SOGIE.

	SO Conversion Attempts (n = 598)	GIE Conversion Attempts (n = 345)
	n/N (%, 95% CI)	
Parent(s), family member(s) or their representatives	406/598 (67.9, 64.0–71.6)	276/345 (80.0, 75.4–84.1)
Friends or acquaintances	139/598 (23.2, 19.9–26.8)	79/345 (22.9, 18.6–27.7)
Members of the clergy or of a religious group	114/598 (19.1, 16.0–22.5)	42/345 (12.2, 8.9–16.1)
Relationship (ex-)partner(s)	82/598 (13.7, 11.1–16.7)	62/345 (18.0, 14.1–22.4)
Healthcare professional(s)	47/598 (7.9, 5.8–10.3)	29/345 (8.4, 5.7–11.9)
doctor or psychiatrist	19/47 (40.4, 26.4–55.2)	20/29 (69.0, 49.2–84.7)
psychologist	24/47 (51.1, 36.1–65.9)	12/29 (41.4, 23.5–61.1)
sexologist	5/47 (10.6, 3.6–23.1)	3/29 (10.3, 2.2–27.4)
nurse	3/47 (6.4, 1.3–17.5)	0/29
other type of therapist, unsure, no recollection	8/47 (17.0, 7.7–30.8)	8/29 (27.6, 12.7–47.2)

*Notes*. Attempts by coworkers, teachers, professors, school staff or unspecified persons were also reported by less than 2% of respondents.

Table 5 reports sociodemographic correlates of SO and GIE conversion attempts. SO conversion attempts were more commonly experienced by respondents who were bisexual, pansexual, and asexual (compared to gay/lesbian), transgender (compared to cisgender), indigenous and racialized (compared to white), and those whose parents were more likely to attend religious services (compared to *never*) and to at least somewhat value religious upbringing (compared to *not at all*). SO conversion attempts were less common among participants who had a college or university education (compared to less than college), who were born in Canada (compared to those born abroad), and who reported an annual household income of over $30,000 CAD (compared to < $30,000 CAD).

**Table 5:** Lifetime prevalence of SOGIE conversion attempts among LGBTQI2+ persons in Quebec, Canada, by participant characteristics.

Variable	SO Conversion Attempts		GIE Conversion Attempts	
	% (95% CI)	Bivariate PR (95% CI)	% (95% CI)	Bivariate PR (95% CI)
**Birth Cohort**				
< 1955	18.2 (12.6–24.9)	Reference	3.7 (1.4–7.8)	Reference
1956–1970	18.8 (15.1–23.1)	1.03 (0.70–1.52)	7.5 (5.1–10.6)	2.04 (0.86–4.81)
1971–1980	21.0 (16.9–25.6)	1.15 (0.79–1.69)	7.7 (5.2–11.0)	2.10 (0.89–4.98)
1981–1990	19.6 (16.9–22.6)	1.08 (0.76–1.54)	9.7 (7.7–12.1)	2.64 (1.17–5.97)
> 1990	20.3 (18.1–22.5)	1.11 (0.79–1.57)	15.7 (13.8–17.8)	4.27 (1.93–9.47)
**Sexual Orientation**				
Gay or Lesbian	18.0 (16.3–19.9)	Reference	6.9 (5.8–8.2)	Reference
Bisexual	21.8 (18.2–25.7)	1.21 (1.00–1.47)	12.9 (10.1–16.2)	1.87 (1.40–2.49)
Queer	22.5 (17.2–28.6)	1.25 (0.96–1.63)	18.0 (13.2–23.7)	2.61 (1.88–3.63)
Pansexual	24.2 (19.6–29.3)	1.34 (1.08–1.67)	24.0 (19.3–29.1)	3.47 (2.67–4.51)
Asexual	28.7 (19.9–39.0)	1.59 (1.14–2.22)	25.8 (17.3–35.9)	3.74 (2.55–5.49)
Other	15.8 (9.1–24.7)	0.88 (0.54–1.41)	20.6 (13.1–30.0)	2.99 (1.95–4.57)
**Gender Modality**				
Cisgender	18.9 (17.4–20.5)	Reference	5.8 (4.9–6.8)	Reference
Trans	24.8 (21.0–28.8)	1.31 (1.10–1.56)	40.5 (36.2–45.0)	7.03 (5.81–8.51)
**Gender Modality & Identity**				
Cisgender Women	21.5 (19.3–23.9)	Reference	7.0 (5.7–8.5)	Reference
Cisgender Men	16.1 (14.1–18.3)	0.75 (0.63–0.88)	4.5 (3.4–5.8)	0.64 (0.46–0.89)
Trans and non-binary AFABs	25.8 (21.2–30.8)	1.20 (0.97–1.48)	40.8 (35.5–46.3)	5.84 (4.61–7.41)
Trans and non-binary AMABs	22.5 (16.3–29.8)	1.04 (0.77–1.42)	39.9 (32.2–48.0)	5.71 (4.33–7.52)
**Intersex Variation**				
No	19.8 (18.4–21.3)	Reference	11.4 (10.3–12.6)	Reference
Yes	38.5 (13.7–68.4)	1.94 (0.97–3.88)	30.8 (9.1–61.4)	2.69 (1.18–6.12)
**Race/Ethnicity**				
White	18.4 (17.0–20.0)	Reference	10.6 (9.4–11.8)	Reference
Indigenous	37.2 (27.5–47.8)	2.02 (1.53–2.66)	28.7 (19.9–39.0)	2.72 (1.94–3.81)
Racialized	29.1 (23.4–35.2)	1.57 (1.27–1.95)	15.4 (11.1–20.5)	1.45 (1.06–1.99)
**Education**				
< College Degree	22.1 (19.9–24.5)	Reference	14.5 (12.7–16.6)	Reference
College/University Degree	17.9 (16.2–19.8)	0.81 (0.70–0.94)	9.0 (7.7–10.5)	0.62 (0.51–0.76)
**Place of Birth**				
Abroad	27.5 (23.4–32.1)	Reference	12.2 (9.2–15.8)	Reference
Canada	18.7 (17.2–20.2)	0.68 (0.57–0.81)	11.4 (10.2–12.7)	0.93 (0.70–1.24)
**Household income**				
< 30,000	25.4 (22.2–28.9)	Reference	16.9 (14.1–19.9)	Reference
30,000–59,999	19.1 (16.3–22.2)	0.75 (0.62–0.91)	13.2 (10.8–15.9)	0.78 (0.61–1.00)
60,000–99,999	18.2 (15.3–21.4)	0.72 (0.58–0.88)	8.9 (6.9–11.4)	0.53 (0.39–0.71)
> 99,999	15.3 (12.7–18.2)	0.60 (0.48–0.75)	5.8 (4.2–7.8)	0.34 (0.24–0.49)
**Parents’ Religious Attendance**				
Never	15.5 (13.5–17.8)	Reference	10.6 (8.9–12.6)	Reference
1–2 times/year	20.1 (17.3–23.3)	1.30 (1.06–1.58)	11.7 (9.5–14.3)	1.10 (0.85–1.44)
> 3 times/year	21.2 (15.8–27.5)	1.36 (1.01–1.84)	12.3 (8.1–17.6)	1.15 (0.77–1.73)
> 1 /month	22.2 (16.9–28.2)	1.43 (1.08–1.89)	9.9 (6.3–14.6)	0.93 (0.60–1.43)
> 1/week	25.5 (22.0–29.2)	1.64 (1.35–2.00)	13.7 (11.0–16.8)	1.29 (0.99–1.69)
**Parents’ Attributed Importance to Religious Upbringing**				
Not at all	15.2 (13.2–17.4)	Reference	11.5 (9.7–13.5)	Reference
Not very	16.5 (13.6–19.8)	1.09 (0.86–1.36)	10.5 (8.1–13.2)	0.91 (0.68–1.21)
Somewhat	20.9 (17.7–24.5)	1.38 (1.12–1.70)	10.3 (8.0–13.1)	0.89 (0.67–1.19)
Very	26.6 (22.4–31.2)	1.75 (1.42–2.16)	12.1 (9.1–15.7)	1.05 (0.77–1.43)
Extremely	36.9 (30.1–44.1)	2.43 (1.93–3.06)	18.1 (13.0–24.3)	1.57 (1.12–2.21)

GIE conversion attempts were more commonly experienced by participants who were born in 1981–1990 and after 1990 (compared to those born before 1955), who reported an intersex variation (compared to endosex respondents), who were bisexual, pansexual, queer, and asexual (compared to gay/lesbian), transgender (compared to cisgender), transmasculine and transfeminine (compared to cisgender women), indigenous and racialized (compared to white), and by participants whose parents extremely valued religious upbringing (compared to *not at all*). GIE conversion attempts were less commonly experienced by respondents with a college or university education (compared to less than college) and by those who reported an annual household income of over $60,000 CAD (compared to < $30,000 CAD).

### Involvement in SOGIE conversion services

Regarding SOGIE conversion services’ involvement (see Table 6), respondents born after 1990 reported the lowest prevalence (2.5%, 95% CI 1.8% to 3.4%), with a gradual increase among older cohorts. Participants born before 1955 were the most likely to have been involved in these services (11.7%, 95% CI, 7.7% to 17.2%) compared to other age cohorts, as were transgender participants (PR = 1.59, 95% CI, 1.10 to 2.30), cisgender men (PR = 1.62, 1.12 to 2.36) and transfeminine participants (PR = 3.28, 1.94 to 5.55) compared to cisgender women, indigenous (PR = 2.10, 95% CI, 1.10 to 4.03) and racialized participants (PR = 2.08, 95% CI, 1.33–3.26) compared to white, and those whose parents attended religious services at least 3 times a year, compared to *never*, and to value religious upbringing *at least somewhat*, compared to *not at all*).

**Table 6:** Lifetime involvement in SOGIE conversion services among LGBTQI2+ persons in Quebec, Canada, by participant characteristics.

Participant Characteristics	n (%), N = 3,263	Distribution of participant characteristics		Involvement by participant characteristics, % (95% CI)	Bivariate PR (95% CI)
		n (%) among involved persons, N = 145	n (%) among never involved persons, N = 3,118		
**Birth Cohort**					
< 1955	180 (5.5)	21 (14.5)	159 (5.1)	11.7 (7.4–17.2)	4.67 (2.79–7.81)
1956–1970	425 (13.0)	33 (22.8)	392 (12.6)	7.8 (5.4–10.7)	3.11 (1.96–4.92)
1971–1980	387 (11.9)	21 (14.5)	366 (11.8)	5.4 (3.4–8.2)	2.17 (1.28–3.67)
1981–1990	829 (25.4)	34 (23.5)	795 (25.5)	4.1 (2.9–5.7)	1.64 (1.03–2.60)
> 1990	1,440 (44.2)	36 (24.8)	1404 (45.1)	2.5 (1.8–3.4)	Reference
**Sexual Orientation**					
Homosexual, gay or lesbian	1,932 (59.3)	90 (62.1)	1,842 (59.1)	4.7 (3.8–5.7)	Reference
Bisexual	537 (16.5)	24 (16.6)	513 (16.5)	4.5 (2.9–6.6)	0.96 (0.62–1.49)
Queer	243 (7.5)	6 (4.1)	237 (7.6)	2.5 (0.9–5.3)	0.53 (0.23–1.20)
Pansexual	340 (10.4)	14 (9.7)	326 (10.5)	4.1 (2.3–6.8)	0.88 (0.51–1.53)
Asexual	104 (3.2)	4 (2.8)	100 (3.2)	3.8 (1.1–9.6)	0.83 (0.31–2.20)
Other terms	105 (3.2)	7 (4.8)	98(3.2)	6.7 (2.7–13.3)	1.42 (0.68–3.01)
**Gender Modality**					
Cisgender	2,705 (83.0)	110 (75.9)	2,595 (83.3)	4.1 (3.3–4.8)	Reference
Trans	556 (17.0)	35 (24.1)	521 (16.7)	6.3 (4.4–8.7)	1.55 (1.07–2.24)
**Gender Modality & Identity**					
Cisgender Women	1,400 (42.9)	44 (30.3)	1,356 (43.5)	3.1 (2.3–4.2)	Reference
Cisgender Men	1,305 (40.0)	66 (45.5)	1,239 (39.8)	5.1 (3.9–6.4)	1.61 (1.11–2.34)
Trans and non-binary AFABs	379 (11.6)	17 (11.7)	362 (11.6)	4.5 (2.6–7.1)	1.43 (0.82–2.47)
Trans and non-binary AMABs	177 (5.4)	18 (12.4)	159 (5.)	10.2 (6.1–15.6)	3.24 (1.91–5.47)
**Intersex Variation**					
No	3,237 (99.6)	142 (98.6)	3,095 (99.6)	4.4 (3.7–5.2)	Reference
Yes	14 (0.4)	2 (1.4)	12 (0.4)	14.3 (1.2–42.8)	3.26 (0.89–11.87)
**Race/Ethnicity**					
White	2,899 (88.9)	115 (79.3)	2,78 (89.4)	4.0 (3.3–4.7)	Reference
Indigenous	108 (3.3)	9 (6.2)	99 (3.2)	8.3 (3.9–15.2)	2.10 (1.10–4.03)
Racialized participants	254 (7.8)	21 (14.5)	233 (7.5)	8.3 (5.2–12.4)	2.08 (1.33–3.26)
**Education**					
College/University Degree	1,851 (57.1)	77 (53.5)	1,774 (57.2)	4.2 (3.3–5.2)	0.86 (0.63–1.19)
< College Degree	1,392 (42.9)	67 (46.5)	1,325 (42.8)	4.8 (3.8–6.1)	Reference
**Place of Birth**					
Canada	2,826 (86.7)	122 (84.1)	2,704 (86.8)	4.3 (3.6–5.1)	0.81 (0.53–1.26)
Outside of Canada	434 (13.3)	23 (15.9)	411 (13.2)	5.3 (3.5–7.8)	Reference
**Household income**					
< 30,000	744 (25.0)	34 (25.8)	71 (25.0)	4.6 (3.2–6.3)	1.21 (0.74–1.97)
30,000–59,000	768 (25.8)	42 (31.8)	726 (25.6)	5.5 (4.0–7.3)	1.45 (0.91–2.31)
60,000–99,000	721 (24.2)	28 (21.2)	693 (24.4)	3.9 (2.6–5.6)	1.03 (0.61–1.72)
> 99,000	741 (24.9)	28 (21.2)	713 (25.1)	3.8 (2.5–5.4)	Reference
**Parents’ Religious Attendance**					
Never	1,117 (39.0)	28 (22.8)	1,089 (39.7)	2.5 (1.7–3.6)	Reference
Once or twice a year	731 (25.5)	22 (17.9)	709 (25.8)	3.0 (1.9–4.5)	1.20 (0.69–2.08)
At least 3 times a year	205 (7.2)	12 (9.8)	193 (7.0)	5.9 (3.1–10.0)	2.34 (1.21–4.52)
At least once a month	223 (7.8)	11 (8.9)	212 (7.7)	4.9 (2.5–8.7)	1.97 (0.99–3.89)
At least once a week	592 (20.6)	50 (40.7)	542 (19.7)	8.4 (6.3–11.0)	3.37 (2.14–5.29)
**Parents’ Attributed Importance to Religious Upbringing**					
Not at all important	1156 (39.4)	29 (23.2)	1,127 (40.1)	2.5 (1.7–3.6)	Reference
Not very important	587 (20.0)	16 (12.8)	571 (20.3)	2.7 (1.6–4.4)	1.09 (0.59–1.98)
Somewhat important	586 (20.0)	27 (21.6)	559 (19.9)	4.6 (3.1–6.6)	1.84 (1.10–3.07)
Very important	409 (13.9)	24 (19.2)	385 (13.7)	5.9 (3.8–8.6)	2.34 (1.38–3.98)
Extremely important	197 (6.7)	29 (23.2)	168 (6.0)	14.7 (10.1–20.5)	5.87 (3.59–9.60)

***Notes***. Participants who reported having been involved in conversion services to change either or both SO and GIE were merged.

Regarding the most recent involvement in SOGIE conversion services (Table 7) results show a wide range in terms of the age at which it took place, with as early as 2 years old and as late as almost 60 years old (median age = 18 years). Over half of occurrences occurred after 2000, with trans participants being more likely to have experienced such involvement after 2009 (57.1, 95% CI: 36.8 to 75.3) compared to cisgender ones (24.8, 95% CI: 16.0 to 36.3). Most commonly, the services were provided by healthcare professionals (doctors, psychiatrists, psychologists, or sexologists), a member of the clergy, another type of professional (e.g., counselors, therapists, teachers, etc.) or, less commonly, by a relative or a family friend. Multiple service providers were identified, which suggests that multiple persons provided conversion services, or that some of them occupied multiple functions (e.g., both a healthcare professional and a member of the clergy, both a sexologist and physician, etc.).

**Table 7:** Context of the most recent involvement in SOGIE conversion services.

	Total (n = 143–144)	Cisgender LGBQ+ Participants (n = 109–110)	Trans Participants (n = 34–35)
**Age** (years)			
Range	2–59	7–56	2–59
Median (Q_1_- Q_3_)	18.0 (16–25)	18.0 (16–24)	18.0 (15–28)
**Calendar year of the most recent experience** (derived from age)	N (%, 95%CI)				
2010–2020	47 (32.6, 23.8–42.9)	27 (24.8, 16.0–36.3)	20 (57.1, 36.8–75.3)
2000–2009	34 (23.6, 16.0–33.4)	27 (24.8, 16.0–36.3)	7 (20.0, 8.3–40.7)
1990–1999	21 (14.6, 8.7–23.4)	19 (17.4, 10.2–28.2)	2 (5.7, 1.2–23.7)
< 1990	42 (29.2, 20.7–39.3)	36 (33.0, 23.0–44.9)	6 (17.1, 6.6–37.6)
**Service providers** †	N (%, 95%CI)		
Any healthcare professional	73 (51.1, 42.6–59.5)	53 (48.6, 38.9–58.4)	20 (58.8, 40.7–75.4)
Doctor or a psychiatrist	30 (21.0, 14.6–28.6)	19 (17.43, 10.8–25.9)	11 (32.4, 17.4–50.5)
Psychologist	34 (23.8, 17.1–31.6)	28 (25.7, 17.8–34.9)	6 (17.7, 6.8–34.5)
Sexologist	11 (7.7, 3.9–13.4)	6 (5.50, 2.1–11.6)	5 (14.7, 5.0–31.1)
Member of the clergy or of a religious group	39 (27.3, 20.2–35.4)	34 (31.2, 22.7–40.8)	5 (14.7, 5.0–31.1)
Other type of provider (counselors, therapists, teachers, etc.)	20 (14.0, 8.8–20.8)	14 (12.8, 7.2–20.6)	6 (17.7, 6.8–34.5)
Family Friend or Relative	6 (4.2, 1.6–8.9)	4 (3.67, 1.0–9.1)	2 (5.9, 0.7–19.7)
**Goals of the service** (*somewhat* or *a lot*)	N (%, 95%CI)		
Among respondents with any SO or GIE conversion experience			
Change gender expression	44 (30.6, 23.2–38.8)	23 (21.1, 13.9–30.0)	21 (60.0, 42.1–76.1)
Among respondents with GIE conversion experience	n = 30–31	n = 8	n = 22–23
Prevent from being or becoming trans	16 (51.6, 33.1–69.9)	1 (12.5, 0.03–52.7)	15 (65.2, 42.7–83.6)
Change gender identity/ to become cisgender	17 (56.7, 37.4–74.5)	2 (25.0, 3.2–65.1)	15 (68.2, 45.1–86.1)
Among respondents with SO conversion experience	n = 112–113	n = 100–101	n = 12
Prevent being or becoming gay, lesbian or bisexual	70 (61.9, 52.3–70.9)	63 (62.4, 52.2–71.8)	7 (58.3, 27.2–84.8)
Change sexual orientation to become heterosexual	79 (70.5, 61.2–76.8)	69 (69.0, 59.0–77.9)	10 (83.3, 51.6–97.9)

^†^ Categories are not mutually exclusive.

Among cisgender participants, conversion services’ main goals were to make them heterosexual (69.0%) or to prevent them from being gay, lesbian, or bisexual (62.4%). Among trans participants, the most reported goals were to make them heterosexual (83%), change their gender identity (68.2%) or their gender expression (60.0%), or to prevent them from being or becoming transgender (65.2%).

Among respondents who were involved in SO conversion services, about 52% consented themselves, of whom only 55% were clearly aware of the services’ objectives (see Table 8). About 48% reported that their parents (or a family member) consented on their behalf, with over 60% of them indicating that the family member(s) did so with clear awareness of the services’ objectives. An additional one-fifth of participants who were involved in SO conversion services reported that someone other than family consented on their behalf, being cognizant of the services’ objectives in over 80% of cases. The most frequently endorsed reasons for using such services were that they thought it would be easier for them and for their future if they tried, and that they could not say no to the person or people who suggested it.

**Table 8:** Informed consent to and reasons for SOGIE conversion service involvement.

	SO Conversion Services (n = 106–113)	GIE Conversion Services (n = 37–41)
Person(s) who consented	N (%, 95%CI)	
Participants	57/110 (51.8, 42.1–61.5)	23/41 (56.1, 39.8–71.5)
Awareness of the services’ objectives = Yes	31/56 (55.4, 41.5–68.7)	7/23 (30.4, 13.2–52.9)
Participants’ parents or family	51/107 (47.7, 37.9–57.5)	15/38 (39.5, 24.0–56.6)
Awareness of the services’ objectives = Yes	28/45 (62.2, 44.5–77.2)	6/15 (40.0, 16.9–68.7)
Someone else^1^	24/106 (22.6, 15.1–31.8)	8/37 (21.6, 9.8–38.2)
Awareness of the services’ objectives = Yes	30/37 (81.1, 64.8–92.0)	5/8 (62.5, 24.5–91.5)
Reasons to use these services^2^	M (SD)	
Thought it would be easier for them and for their future if they tried	1.67 (0.12)	1.89 (0.19)
Wanted to become heterosexual or cisgender	1.40 (0.12)	1.27 (0.20)
Afraid of negative consequences in case of refusal (e.g., family rejection, refusal of care, termination of treatment)	1.39 (0.13)	1.70 (0.21)
Felt that their loved ones would be happy if they did it	1.41 (0.12)	1.65 (0.20)
Could not say no to the person or people who suggested it	1.52 (0.12)	1.59 (0.22)
Were convinced it was a good idea to try	1.28 (0.12)	1.54 (0.21)

*Notes*. M, mean; SD, standard deviation.

^1^ Includes: physician, member of the clergy, psychologist, friends, school staff (principal, teacher).

^2^ Ranging from 0 (*Totally false*) to 3 (*Totally True*).

Approximately 56% of respondents having been involved in GIE conversion services consented themselves, 30% of whom were cognizant of these services’ conversion goals. Forty percent reported that their family consented for them, with clear awareness of the services’ objectives in 40% of cases. One-fifth of the participants who were involved in GIE conversion services reported that someone other than family consented on their behalf (e.g., physician, member of the clergy, psychologist, friends, school staff, etc.), close to 63% of whom were clearly aware of the services’ objectives. The main reasons endorsed for using these services were to make their lives and futures easier, to please their loved ones, and because they feared negative consequences in case of refusal (e.g., family rejection, refusal of care, termination of treatment, etc.).

## Discussion

This study is the first to report data on lifetime exposure to various forms of SOGIE conversion efforts across all gender identities and modalities and sexual orientation groups in Canada. We used data from a large community-based survey to investigate SOGIE prevalence and correlates among LGBTQI2+ people in the province of Quebec. This study revealed that 4.4% of the sample used SOGIE conversion services, with higher prevalence rates among trans participants (PR = 1.59, 95% CI, 1.10 to 2.30). The overall rate of conversion service involvement was close to those reported for Quebecois MSM in the Canadian *Sex Now* survey (4.2%, [4]; 6.8%; [3]). In the current study, conversion services involvement among transgender participants (6.3%) was lower than that reported in the US Transgender Survey (i.e., 14%; [6]).

SOGIE modification attempts were far more prevalent than conversion services involvement in the current LGBTQI2+ sample (25%), particularly among trans participants who were about 7 times more likely to report so compared to cisgender participants. In comparison, Salway et al. [5] report a lower prevalence among sexual-minority men (15% in Quebec, 20% across Canada). Disparities in these estimates may reflect variations in the wording of the phenomenon, such as services involvement, sustained efforts [5], conversion therapy [1, 7, 13], reparative therapy [7], attempts or efforts [3], treatment or cure [8], or sexual repair/reorientation [4], as well as geographical variations in the conversion services offered and in the societal attitudes toward sexual and gender diversity across the US and Canada, and in Quebec more specifically.

### Correlates of conversion services involvement

It should be noted that licensed healthcare providers were responsible for about half of the current sample’s most recent conversion experiences, and members of the clergy or of a religious group, for about one-third. Over half of participants reported having consented to SOGIE conversion services. These services’ objectives, however, were clear to only 55% of those who engaged in SO conversion and to 30% of those who engaged in GIE conversion, which suggests that the goal of conversion became known to these participants only once after being involved in the process. These numbers are higher than those of a previous study that included participants from over 100 countries and showed that only one-fourth of the sample have sought conversion services on their own, while the rest of respondents declared that the decision was largely outside of their control [18].

The results also show that other persons were also involved to varying degrees in these decisions (parents, extended family, religious congregation members, or school personnel, including private school personnel, which are assumed to be religious), and that these individuals were more likely than the participants to have been aware of the services’ conversion goals. Overall, conversion services involvement among Quebec LGBTQI2+ persons likely resulted from concerted efforts from their immediate environment. Given that the most frequently endorsed reasons to consent to or comply with these services were the wish for a better future and the fear of rejection, it is also likely that these individuals were swayed by ambient hostility toward sexual and gender diversity, leading them to believe that SOGIE conversion was their best option. The results further revealed that a high percentage of participants, parents, and family members were unaware of conversion services’ goals, suggesting that they may involve deception or manipulation, especially in relation to GIE conversion services. To increase power when exploring for correlates, we merged participants who reported having accessed any SO or GIE conversion services. This decision was supported by our finding that response patterns concerning both services were similar. Contrary to Salway et al. [3] who found a greater exposure to conversion therapy practices among younger generations of Canadian MSM, we observed a birth cohort effect regarding the accessing of conversion services, with older cohorts being more likely than younger ones to report having done so. This pattern may reflect changes in societal attitudes and professional regulations that oppose SO conversion practices, and only more recently, GIE conversion practices.

Accessing SOGIE conversion services was also more commonly reported by indigenous and racialized participants, as well as by those from more religious families. These findings support those of previous studies, and suggests that these groups may endorse more conservative values and stricter sexuality and gender norms (often imported from a colonial past; see Barker [27]), which contribute to create a hostile climate toward sexual and gender diversity and to increase the likelihood of seeking SOGIE conversion services [15]. While there were no variations between sexual orientation groups regarding SOGIE conversion services involvement, we found that transgender persons and those assigned male at birth (cisgender, transgender, or non-binary) presented increased risk. This points to a lower threshold for gender (non-)conformity tolerance toward persons assigned male at birth. Contrary to Salway et al. [4], low-income participants were not more likely to have been involved in SOGIE conversion services than those with higher incomes.

### Correlates of conversion attempts

While our findings regarding SOGIE conversion attempts also confirm the role of sociocultural context, differences between SO and GIE conversion attempts are noteworthy. While SO conversion efforts occurred in similar proportions across birth cohorts, GIE attempts were mainly reported by younger generations. This could reflect socially and politically conservative reactions to increased consultations regarding GIE variations among younger generations [28]. Such conversion attempts could also be due to more recent cohorts of non-binary and transgender persons coming out earlier due to increasing trans visibility, and while still living in their parents’ homes, which can make them more vulnerable to family pressure and other cisnormative influences.

Both SO and GIE conversion attempts were more commonly reported by less educated and lower income participants, while SO conversion attempts more specifically were more common among those who were from more religious households and who were born outside Canada. These results confirm previous findings about the key role of geographical and socioeconomic factors in creating a social or family context that is hostile to sexual and gender diversity. As Salway et al. [4] suggested, it is possible that the association between ethnicity and racialized status and SO conversion efforts may be at least partially explained by the mediating effect of socioeconomic factors. Yet, as exposure to SOGIE conversion likely happened before the income and education level measured at the time of the study, it is more likely that exposure to SOGIE conversion efforts has negatively impacted the socioeconomic trajectory, a hypothesis that is also suggested by other authors [8].

That SOGIE conversion attempts were more commonly reported by individuals who identified as other than gay or lesbian can reflect the greater acceptance and recognition of gay and lesbian persons, while other sexual identities remain misunderstood (e.g., bisexuality and pansexuality) or conceptualized as sexual disorders (e.g., asexuality). Results revealed how cisnormativity can also affect intersex persons, who were more likely than their endosex counterparts to have been exposed to GIE, but not SO, conversion attempts. Moreover, our results showed that, compared to their cisgender counterparts, transgender persons were more exposed to both SO and GIE conversion attempts. Their higher exposure to SO conversion efforts might reflect how gender (non)conformity is often taken as a sign of non-heterosexuality. Unlike conversion services, there were no significant differences in the rates of conversion attempts between trans people assigned male and assigned female at birth.

### Strengths and limitations

This study is the first to examine exposure to both SO and GIE conversion attempts and conversion services involvement across multiple sexual orientation groups and gender identities and modalities in a large sample. Yet, this work also has some limitations. First, its cross-sectional, retrospective design is subject to recall bias and prevents any causal inferences. Second, as for any self-selected, non-probabilistic sampling, it is likely that the LGBTQI2+ persons who volunteered to participate are different from those who did not. While we used multiple, diversified recruitment strategies, the results cannot be generalized beyond the present sample. Third, as the SOGIE conversion experiences were elicited using non-validated self-reports, our indicators may not have accurately captured their prevalence.

Despite these weaknesses, this study provides a unique overview of Quebec’s LGBTQI2+ populations’ SOGIE conversion experiences, including women’s (cisgender and transgender). The results highlight that while conversion services involvement was more common among older generations, conversion attempts were more common among younger ones. Our findings also show the increased vulnerability to conversion attempts and service involvement among participants with religious upbringing, indigenous persons and people of colour, intersex, transgender, non-binary and asexual persons, as well as those who did not have a monosexual sexual orientation (bisexual, pansexual).

To protect LGBTQI2+ persons from such attempts and practices, legal bans on conversion practices are an important step as they send a strong message about their unethical and harmful nature. However, they will not be insular to faith-based practices and they will be insufficient to eliminate pressures and practices covertly operating under the guise of exploration. Professionals’ ongoing commitment is sorely needed, professional associations must expand their statements regarding sexual orientation and gender identity and expression practices, and healthcare providers need adequate training in LGBTQI2+ affirmative approaches. Religious counselors should also address the religious beliefs and cisheterosexist assumptions underlying their spiritual guidance or clinical practice. Addressing such biases does not imply deconstructing their religious beliefs, but rather exploring how their faith can impact their clinical practice [29, 30]. Moreover, as families play a key role in pressuring children into conversion practices, they need to be supported and counseled in the acceptance of their LGBTQIA+ children. More studies are needed to better understand parental and family characteristics associated with heterosexism and cissexism.

## Supporting information

S1 File Alternative language abstract. (DOCX)

## References

[1] The Trevor Project. National Survey on LGBTQ Mental Health. [Internet]. 2019 [cited 2021 Apr 21]. https://www.thetrevorproject.org/survey-2019/

[2] Ashley F. Model Law–Prohibiting Reparative Practices [Internet]. McGill University; 2020. https://papers.ssrn.com/sol3/papers.cfm?abstract_id=3398402

[3] Salway T, Juwono S, Klassen B, Ferlatte O, Ablona A, Pruden H, et al. Experiences with sexual orientation and gender identity conversion therapy practices among sexual minority men in Canada, 2019–2020. Yang XY, editor. PLoS ONE. 2021 Jun 3;16(6):e0252539. doi: 10.1371/journal.pone.0252539 34081740 PMC8174694

[4] Salway T, Ferlatte O, Gesink D, Lachowsky NJ. Prevalence of Exposure to Sexual Orientation Change Efforts and Associated Sociodemographic Characteristics and Psychosocial Health Outcomes among Canadian Sexual Minority Men. Can J Psychiatry. 2020 Jul 1;65(7):502–9. doi: 10.1177/0706743720902629 31984758 PMC7298582

[5] Salway T, Lachowsky N, Kwag M. Conversion Therapy & SOGIECE in Canada—Sex Now Survey results reveal prevalence of change efforts. Vancouver: Community-based Research Centre. [Internet]. 2020. https://www.cbrc.net/sex_now_survey_results_reveal_prevalence_of_change_efforts

[6] Turban JL, Beckwith N, Reisner SL, Keuroghlian AS. Association Between Recalled Exposure to Gender Identity Conversion Efforts and Psychological Distress and Suicide Attempts Among Transgender Adults. JAMA Psychiatry. 2020 Jan 1;77(1):68–76. doi: 10.1001/jamapsychiatry.2019.2285 31509158 PMC6739904

[7] Green AE, Price-Feeney M, Dorison SH, Pick CJ. Self-Reported Conversion Efforts and Suicidality Among US LGBTQ Youths and Young Adults, 2018. Am J Public Health. 2020 Jun 18;110(8):1221–7. doi: 10.2105/AJPH.2020.305701 32552019 PMC7349447

[8] Ryan C, Toomey RB, Diaz RM, Russell ST. Parent-Initiated Sexual Orientation Change Efforts With LGBT Adolescents: Implications for Young Adult Mental Health and Adjustment. Journal of Homosexuality. 2020 Jan 28;67(2):159–73. doi: 10.1080/00918369.2018.1538407 30403564 PMC10371222

[9] Drescher J, Schwartz A, Casoy F, McIntosh CA, Hurley B, Ashley K, et al. The Growing Regulation of Conversion Therapy. Journal of Medical Regulation. 2016 Jan 1;102(2):7–12. 27754500 PMC5040471

[10] SQ 2020, c 28 | An Act to protect persons from conversion therapy provided to change their sexual orientation, gender identity or gender expression [Internet]. CanLII; [cited 2021 Dec 6]. https://www.canlii.org/en/qc/laws/astat/sq-2020-c-28/latest/sq-2020-c-28.html

[11] Government Bill (House of Commons) C-4 (44–1)—First Reading—An Act to amend the Criminal Code (conversion therapy)—Parliament of Canada [Internet]. [cited 2021 Dec 6]. https://www.parl.ca/DocumentViewer/en/44-1/bill/C-4/first-reading

[12] The Trans PULSE Canada Team. QuickStat #1 –Conversion Therapy. 2019-12-20. [Internet]. 2019. https://transpulsecanada.ca/research-type/quickstats/

[13] Meanley S, Haberlen SA, Okafor CN, Brown A, Brennan-Ing M, Ware D, et al. Lifetime Exposure to Conversion Therapy and Psychosocial Health Among Midlife and Older Adult Men Who Have Sex With Men. The Gerontologist. 2020 Oct 1;60(7):1291–302. doi: 10.1093/geront/gnaa069 32556123 PMC8189432

[14] Ashley F. Homophobia, conversion therapy, and care models for trans youth: defending the gender-affirmative approach. Journal of LGBT Youth. 2020 Oct 1;17(4):361–83.

[15] Kanbur N. Internalized Homophobia in Adolescents: Is it really about Culture or Religion? J Can Acad Child Adolesc Psychiatry. 2020 May;29(2):124–6. 32405314 PMC7213920

[16] Flores AR, Mallory C, Conron KJ. Public attitudes about emergent issues in LGBTQ rights: Conversion therapy and religious refusals. Research & Politics. 2020 Oct 1;7(4):2053168020966874.

[17] Richter BEJ, Lindahl KM, Malik NM. Examining ethnic differences in parental rejection of LGB youth sexual identity. Journal of Family Psychology. 2017;31(2):244–9. doi: 10.1037/fam0000235 27571323 PMC5328793

[18] Adamson T, Wallach S, Garner A, Hanley M, Howell S. The Global State of Conversion Therapy—A Preliminary Report and Current Evidence Brief [Internet]. SocArXiv; 2020 [cited 2021 Apr 21]. Available from: https://osf.io/preprints/socarxiv/9ew78/

[19] Turban JL, King D, Reisner SL, Keuroghlian AS. Psychological Attempts to Change a Person’s Gender Identity From Transgender to Cisgender: Estimated Prevalence Across US States, 2015. Am J Public Health. 2019 Oct;109(10):1452–4. doi: 10.2105/AJPH.2019.305237 31415210 PMC6727306

[20] Brotto LA, Yule M. Asexuality: Sexual Orientation, Paraphilia, Sexual Dysfunction, or None of the Above? Arch Sex Behav. 2017 Apr;46(3):619–27. doi: 10.1007/s10508-016-0802-7 27542079

[21] Brown LD, Cai TT, DasGupta A. Interval Estimation for a Binomial Proportion. Statistical Science. 2001;16(2):101–17.

[22] Goodman LA. On Simultaneous Confidence Intervals for Multinomial Proportions. Technometrics. 1965;7(2):247–54.

[23] Zou G. A modified poisson regression approach to prospective studies with binary data. Am J Epidemiol. 2004 Apr 1;159(7):702–6. doi: 10.1093/aje/kwh090 15033648

[24] Rea LM, Parker RA. Designing and Conducting Survey Research: A Comprehensive Guide. John Wiley & Sons; 2014. 352 p.

[25] Kotrlik JW, Williams HA, Jabor MK. Reporting and Interpreting Effect Size in Quantitative Agricultural Education Research. Journal of Agricultural Education. 2011;52(1):132–42.

[26] Madley-Dowd P, Hughes R, Tilling K, Heron J. The proportion of missing data should not be used to guide decisions on multiple imputation. Journal of Clinical Epidemiology. 2019 Jun;110:63–73. doi: 10.1016/j.jclinepi.2019.02.016 30878639 PMC6547017

[27] Barker J. Critically sovereign: Indigenous gender, sexuality, and feminist studies. [Internet]. Critically Sovereign. Duke University Press; 2020 [cited 2021 May 28]. https://www.degruyter.com/document/doi/10.1515/9780822373162/html

[28] Brady CE, Ernst MM. Pediatric Gender Identity: Consultation on Matters of Identity, Transgender Concerns, and Disorders/Differences of Sex Development. In: Carter BD, Kullgren KA, editors. Clinical Handbook of Psychological Consultation in Pediatric Medical Settings [Internet]. Cham: Springer International Publishing; 2020 [cited 2021 Dec 6]. p. 439–49. (Issues in Clinical Child Psychology). 10.1007/978-3-030-35598-2_33

[29] McGeorge CR, Coburn KO, Walsdorf AA. Christian Mainline Protestant Pastors’ Beliefs About the Practice of Conversion Therapy: Reflections for Family Therapists. Journal of Marital and Family Therapy. 2021;47(3):698–712. doi: 10.1111/jmft.12447 32761630

[30] Whitman JS, Bidell MP. Affirmative lesbian, gay, and bisexual counselor education and religious beliefs: How do we bridge the gap? Journal of Counseling & development. 2014;92(2):162–9.

