# Supplementary material for: Sexual orientation and gender identity and expression conversion exposure and their correlates among LGBTQI2+ persons in Québec, Canada
Source: PLoS One. 2022 Apr 6;17(4):e0265580. doi: 10.1371/journal.pone.0265580 (PMC8986006; doi:10.1371/journal.pone.0265580)
Supplement: S1 File — (DOCX) [file pone.0265580.s001.docx]

**Résumé**

**Contexte**

Malgré une plus grande acceptation de la diversité sexuelle et de genre et le consensus scientifique selon lequel l'attirance pour le même genre, l'expression créative du genre et la transidentité ne sont pas des maladies mentales, les personnes LGBTQI2+ se font encore souvent dire qu'elles peuvent ou doivent changer leur orientation sexuelle, leur identité de genre ou leur expression de genre (OSIEG). L'objectif de cette étude est de décrire la prévalence des efforts de conversion de l’OSIEG, y compris leurs corrélats sociodémographiques, parmi les personnes LGBTQI2+.

**Méthodes**

En utilisant un échantillonnage communautaire, nous avons évalué les tentatives de conversion de l’OSIEG et l'implication dans les services de conversion de 3 261 personnes LGBTQI2+ âgées de 18 ans et plus au Québec, Canada.

**Résultats**

Un quart des participant.e.s ont fait l'objet de tentatives de conversion de l’OSIEG et moins de 5% ont été impliqués dans des services de conversion. Parmi les répondants qui ont été impliqué dans des services de conversion, plus de la moitié a consenti à ces services. Toutefois, les objectifs de conversion de l’OS ou de l’IEG n’étaient clairs que pour 55% et 30% d’entre eux respectivement. Les résultats suggèrent que la famille joue un rôle clé dans les tentatives de conversion SOGIE et l’implication dans des services. Les personnes autochtones et de couleur, les personnes intersexuées, transgenres, non-binaires et asexuelles, ainsi que celles dont l'orientation sexuelle n'est pas monosexuelle (c'est-à-dire bisexuelles, pansexuelles) étaient plus susceptibles d'avoir été exposées à des tentatives de conversion et d'avoir été impliquées dans des services de conversion.

**Conclusions :**

Cette étude montre que la prévalence des efforts de conversion est élevée, notamment en termes de tentatives. Les interventions visant à protéger les personnes LGBTQI2+ de telles tentatives devraient se concentrer sur le soutien aux familles qui doivent être conseillées dans l'acceptation de la diversité sexuelle et de genre, et les professionnels de santé doivent être formés de manière adéquate aux approches affirmatives LGBTQI2+.
